# Differences in Perceptions of Patient Safety Culture between Charge and Noncharge Nurses: Implications for Effectiveness Outcomes Research

**DOI:** 10.1155/2012/847626

**Published:** 2012-03-22

**Authors:** Deleise Wilson, Richard W. Redman, AkkeNeel Talsma, Michelle Aebersold

**Affiliations:** University of Michigan School of Nursing, Ann Arbor, 48109 MI, USA

## Abstract

The implementation of evidence-based practice guidelines can be influenced by nurses' perceptions of the organizational safety culture. Shift-by-shift management of each nursing unit is designated to a subset of staff nurses (charge nurses), whom are often recruited as champions for change. The findings indicate that compared to charge nurses, noncharge nurses were more positive about overall perceptions of safety (*P* = .05) and teamwork (*P* < .05). Among charge nurses, significant differences were observed based on the number of years' experience in charge: perception of teamwork within units [*F*(3, 365) = 3.52, *P* < .01]; overall perceptions of safety, [*F*(3, 365) = 4.20, *P* < .05]; safety grade for work area [*F*(3, 360) = 2.61, *P* < .05]; number of events reported within the last month [*F*(3, 362) = 3.49, *P* < .05]. These findings provide important insights to organizational contextual factors that may impact effectiveness outcomes research in the future.

## 1. Introduction

With the increasing emphasis in the efficient delivery of healthcare, healthcare organizations are investing in effectiveness outcomes research to improve patient outcomes. However, the uptake and implementation of evidence-based clinical practice guidelines are influenced by contextual factors such as leadership support and use of change champions [1–3] and personnel perceptions of patient safety [4]. Within acute care settings, nurses' perceptions of patient safety cultures and attitudes towards new practice guidelines are very critical for predicting the use of research evidence and new guidelines [5, 6]. What is known about nurses' perceptions of patient safety culture has been reviewed in comparison with interdisciplinary team members [7–9] and across ranks such as staff nurses versus nurse managers [10]. Yet, staff nurses are not a homogenous group. In most acute care settings for each nursing unit, the management of each shift is designated to a nurse who then leads other staff nurses on that shift. The shift-by-shift leaders may be known as charge nurses, or assistant nurse managers [11, 12] and are often used as champions for change [13, 14]. Since nurses are very pivotal to the implementation of safety guidelines, it is critical to have a deeper understanding of how these two groups of nurses, charge and noncharge nurses, perceive patient safety cultures. The purpose of this paper was to compare the perceptions of nursing units' safety culture between charge nurses and staff nurses. For this study, the charge nurse is defined as a frontline nursing unit leader who makes shift-by-shift decisions about staffing, personnel and unexpected events that impact patient care [15]. In contrast, the noncharge nurse is defined as a staff nurse who is a direct patient care provider and has never had charge nurse experience.

## 2. Background

The creation of reliable healthcare organizations is fundamental for the process of improving patient care [16, 17]. The use of evidence-based practice guidelines has become widespread as one of several methods healthcare organizations seek to establish safe and reliable practice environments [18, 19]. Notwithstanding, there are many organizational barriers that limit the implementation of practice guidelines [20, 21]. Ricart et al. [22] found that nurses' fear of potential harm to patients contributed to nonadherence to evidence-based guidelines for the prevention of ventilator-associated pneumonia. Doherty [23] found that the lack of regular nursing staff educational meetings was a barrier to the implementation of adult asthma guidelines in the emergency room. Similarly, in the examination of the use of research in nursing organizations, Estabrooks et al. [5] stated that work and communication patterns characteristic of the nurses and the types of decision making processes predicted variability across organizations. Likewise, the positive perceptions of patient safety culture were associated with greater use of research findings and lower in adverse patient outcomes [2, 3, 24].

However, perceptions of patient safety culture vary across disciplines, healthcare settings, and professional ranks [25, 26]. Notably, leaders are often associated with having more positive perceptions of the safety culture than frontline workers, and managers and physicians generally reported higher levels of positive perceptions of safety as compared to staff nurses [27]. Singer et al. [28] found that among nurses, work experience and work position were significantly associated with perceptions of the patient safety culture. There were more positive reports from nurses who worked on a unit or hospital for more than 10 years, while Kim et al. [10] also found distinctions in perceptions of patient safety culture between staff nurses and managers among healthcare workers, but we would propose that this does not go far enough to examine potential differences between staff nurses and charge nurses.

 At the nursing unit-level staff nurses function as either charge or noncharge nurses. Charge nurses generally function as shift-by-shift leaders of nursing units whose duties may vary within and across organizations [29, 30]. Staff nurses tend to be recently hired, mainly provide direct patient care and are supervised by charge nurses [15, 31]. In the implementation of evidence-based practice initiatives, the nurses recruited as change champions can be either charge or noncharge nurses [32, 33].

Similar to findings about other contextual factors influencing effective outcomes research, the impact of opinion leaders is also multifaceted [6, 34]. Curran's [35] study of opinions leaders indicated that the success of an opinion leader in leading change was influenced by acceptance of the role, developmental level of the social networks within organizations clarity of role expectations and perceptions of organizational context. Although positive perceptions of patient safety culture have influenced increased use of practice guidelines as reported by Estabrooks et al. [5] and Cummings et al. [2], there may be challenges to smooth implementation when confronted with differences in perceptions of organizational context experienced by change champions [36, 37]. To disentangle the effects of nurses' perceptions of patient safety culture on the use of evidence-based practice guidelines, it may be necessary to determine whether differences in perceptions do exist between charge and noncharge nurses. With this need, this paper was aimed at exploring the differences in perceptions of safety culture between charge and noncharge nurses.

## 3. Methods

### 3.1. Design and Participants

This study used a descriptive, correlational and cross-sectional design to examine the differences in the perceptions of patient safety culture among registered nurses working in 12 adult medical surgical units at a large academic medical center in the Midwest. There were 710 registered nurses working in the 12 units at the time of the study. To be included in this study, the nurses had to have at least six months experience on their current unit and were supervised by a charge nurse or worked as charge nurse. LPNs and nurse managers were excluded from the study.

### 3.2. Data Collection

Following the approval of the institutional review board (IRB) of the medical center, a modified Dillman method was used to recruit nurses [38]. The design involved engaging the study participants in the following manner: (1) questionnaires in large manila envelopes were placed in staff nurses' unit mailboxes; (2) 1-2 weeks after the study began, a thank you postcard was placed in the mailboxes to express appreciation for completion or as a reminder if the questionnaire had not been returned; (3) 3-4 weeks after, a thank you postcard was placed in mailboxes to express appreciation for completion of survey or as a gentle reminder if the questionnaire had not been returned. Completed surveys were placed in sealed drop boxes located within each nursing unit and sequentially numbered as they were returned. A total of 710 surveys were distributed. Over a 3-month period, 381 nurses returned completed questionnaires and signed consent forms, which yielded a response rate of 54%. Six of the 381 questionnaires were not used in the analyses on account of missing data that exceeded 10% of the total items in the study. The final sample, therefore, consisted of 375 respondents representing 53% of the total possible registered nurses who met the inclusion criteria.

### 3.3. Measures

The independent variables were charge nurse experience (no charge and some charge), percentage of shifts worked incharge in the past month (<25% and >25%), and number of years as charge nurse on current unit (none, less than 1 year, 1 to 5 years, and more than 5 years). Shift worked was a categorical variable with three options: permanent day, permanent night, and rotating shift. The demographic variables for the study were level of highest degree, length of time in current unit. The educational level options were (1) diploma and associate's degrees; (2) baccalaureate degree; (3) master's degree. Length of time in current unit response categories were (1) less than 1 year; (2) 1 to 5 years; (3) more than 5 years.

There were four dependent variables in the study, namely; overall perceptions of patient safety, number of events reported, teamwork within units, and safety grade. These dependent variables are four of the eleven subscales of the AHRQ Hospital Survey on Patient Safety Culture survey [39]. Researchers have found the AHRQ Hospital Survey on Patient Survey Culture to be reliable ranging from .72 to .84 with the exception of the staffing dimension (.63) [7]. In this study, the Cronbach alpha for overall perceptions of safety was .70, and teamwork within units was .80 [7]. Safety grade and number of events reported were single items.

### 3.4. Data Analysis

The statistical package for the social sciences (SPSS) software version 18.0.3 was used for analyses of the data. At the completion of data entry, there were fewer than 5% of missing items. Following the guidelines of McKnight et al. [40], this is below the 10% threshold. Therefore, the items were not deleted and were included in data analysis. Mean substitution done to impute the values for the missing items. *t*-tests were conducted to test the hypothesis that there were differences in patient safety culture between nurses with no charge and some charge experience. Pearson's chi-square test was utilized to test the relationship between percentages of shifts in charge during the past month. ANOVA technique was utilized to examine differences in the perceptions of patient safety among nurses with varying percentages of shifts in charge and number of years as charge nurse during the past month.

## 4. Results

The descriptive characteristics of the study sample can be found in Tables 1 and 2. The sample of registered nurses consisted of 215 nurses with some charge experience and 159 without charge experience. Six out of ten of the nurses with no charge experience had a bachelor's degree as compared to five out of ten of those with some charge experience. The nurses who were never in charge worked mainly during the rotating shifts (46%) with the least (17%) working the permanent day shift and 37% working the permanent night shift. A somewhat opposite pattern was noted in the nurses with some charge experience: 47% worked during the day; 31% at night; 22% percent worked as shift rotators. Of the nurses who were in charge, 47% worked on the current unit for more than six years compared to 12% of the staff nurses. Only 31% of the nurses functioned in the charge role for greater than twenty-five percent of shifts worked, while 25% were in charge for less than twenty-five percent of the shift worked, and the remaining 44% were never in charge. Interestedly, only 6% of the charge nurses self-identified as being permanent in the role in that they were in charge for 75% or greater of shifts worked. The educational preparation for those who were charge nurses was captured by number of shifts for shadow-charge orientation. Eight percent of the charge nurses stated they had no shadow-charge orientation. The majority (63%) of charge nurses had one to two shifts, while 29% had 3 or more shifts of shadow charge experience.

A two-tailed *t*-test for independent groups was used to test the hypothesis that the nurses with no charge and some charge experience will have differences in perception of safety. Significant differences were observed with two dimensions of the patient safety culture. The *t*-test revealed that for nurses with no charge experience the mean (3.46) for overall perception of safety was significantly higher than for the nurses with some charge experience (3.27), [*t*(374) = 2.86, *P* = .005]. Consistent with that finding, for the dimension number of events reported within a 12-month period, the nurses with some charge had a higher mean (2.31) than nurses with no charge experience (2.06), [*t*(368) = −3.35, *P* = .001]. These findings are summarized in Table 3.

The nurses with no charge experience reported fewer events. No events were reported by 21% of the nurses with some charge experience versus 14% of those with no charge experience. Of those who reported 1 to 2 events, 52% were reported by nurses with no charge experience as compared to 42% with some charge experience. As the number of events increased to 3 to 21 events, the nurses with some charge experience (45%) reported more events versus 27% of the nurses with no charge experience.

The Pearson's chi-square test was utilized to test the relationship between percentage of shifts in charge during the past month and number of events reported in the past month. As shown in Table 4, fifty-two percent of the nurses with no charge experience reported 1 to 2 events; 20% reported no events; 28% reported 3 to 21 events. The nurses with no charge experience were almost equally divided between no events (20%) and 3 to 21 events (28%). The nurses with less than 25% of the shifts worked had the highest percent (47%) reporting 1 to 2 events, which is similar to the nurses with no charge experience. Moreover, 41% reported 3 to 21 events and 12% reported no events. Of the nurses who were in charge for more than twenty-five percent of shifts worked, 37% reported 1 to 2 events; 48% reported 3 to 21 events; 16% reported no events.

The nurses with no experience (20%) had a higher percentage of reporting no events as compared to the nurses with less than twenty-five percent of shifts in charge (12%) and more than twenty-five percent of shifts in charge (16%). The nurses who were in charge for greater than twenty-five percent of shifts worked reported 3–21 events three times more than they reported no events. In the category of 1 to 2 events, there was a higher percentage of nurses with no charge experience (52%) reporting as compared to the nurses with some experience. The nurses with some charge experience tended to report more events.

Utilizing ANOVA technique, differences in the perceptions of patient safety among nurses with varying percentages of shifts in charge during the past month were significant differences in overall perception of safety, [*F*(2,369) = 3.27, *P* < .05]. Bonferroni's post hoc test showed that there were differences between nurses with no charge nurse shifts and those with greater than 25% of shifts in charge in the last month.

There were also variations among the number of years as charge nurse for the perceptions of teamwork within units [*F*(3,365) = 3.52, *P* < .01], overall perceptions of safety, [*F*(3,365) = 4.20, *P* < .05], safety grade for work area [*F*(3,360) = 2.61, *P* < .05], and number of events were reported within the last month [*F*(3,362) = 3.49, *P* < .05]. Further analysis using Bonferroni's post hoc tests indicated the differences in perception of patient safety among the nurses with less than one year, one to five years and more than five years as charge nurse for teamwork within hospital units, the nurses with less than one year of experience were more positive than nurses with more than 5 years (*P* < .05). For overall perceptions of safety, the nurses who were never in charge had more positive perceptions of safety than those who were in charge for one to five years for more than 5 years (*P* < .01). The differences in safety grade for work area were between the nurses with no charge, who were more positive than the nurses with more than five years of charge experience (*P* < .05), and for the number of events reported within the last twelve months the nurses who were never in charge were more positive than those with one to five years of charge experience (*P* < .05).

## 5. Discussion

The purpose of the study was to evaluate whether differences in perceptions of safety exist between charge and staff nurses. Differences in perception of patient safety culture between and among charge nurses were established in this study. Specifically, we found that there were differences observed in perceptions of teamwork within the unit; overall perceptions of safety; safety grade for area; number of events reported within the last twelve months according to the number of years as a charge nurse. Nurses with no charge experience had more positive overall perceptions of patient safety, while the nurses with some charge experience had less positive overall perceptions of safety. Charge nurses with one to five or more than five years of experience were less positive about teamwork, overall perceptions of safety, safety grade for work area, and number of events reported. The percentage of shifts worked in charge in the past month provides more information about the differences observed between the charge and noncharge nurses. The results support differences in overall perception of safety between the nurses with greater than 25% of shifts in charge and with no shifts in charge. Chi-square tests for the patient safety grade for work area and charge nurse experience revealed no significant findings.

These findings run counter to the results from previous studies which indicate that there are less positive perceptions of patient safety by frontline nurses in general [7, 8]. Kim et al.'s [10] study about nurses' perceptions of patient safety included 10% (*n* = 86) charge nurses, but they did not report findings that compared charge nurse perceptions of patient safety with those of other groups of nurses. Unlike Kim et al. [10], this current paper focuses on charge nurses as a discrete group. In a previous study, registered nurses in general with more experience and length of time on the unit were more positive about patient safety culture [10]. Other studies about perceptions of patient safety culture included nurses as a monolithic subset among healthcare providers such as physicians, clinical or nonclinical managers, and technicians [26]. In this regard, this current study marks an important departure from other empirical findings about role of leaders in perceptions of patient safety in health care organizations, especially as it pertains to nurses. This, in turn, may have important implications for how these leaders promote implementation of evidence-based practice as well.

In other findings, new graduates tended to make more medication errors [41] and are perceived to contribute more to errors than older, more experienced nurses [42]. Previous studies had also shown that new graduates were less positive about their work environment because they are more stressed adjusting to the work environment [43], emotionally exhausted [44], or overwhelmed [45]. However, the finding in this study that indicated the new graduates were more positive about perceptions of safety may be due in part, to observations that they may not have received adequate education about patient safety [46], may be more focused into developing critical thinking skills or their personal safety practices as against the demands of collective unit responsibility [47].

The differences in safety perceptions between nurses with no and some charge experiences may be explained by the fact that the charge nurses have a broader overview of potential and real safety errors and may be more familiar with the error reporting system or are more aware of the errors occurring on the unit than staff nurses, which influences their perceptions of patient safety adversely. Further, even differences noted among nurses who have charge experience. In a previous study, registered nurses with more experience and length of time on the unit were more positive about patient safety culture [10]. In this study, the more experienced charge nurses were less positive about patient safety culture. This may be indicative of a lack of full expertise by those who are in charge for less than 25% of shifts worked. Therefore, the nurses who move in and out of the charge nurse role and spend more time as a staff nurse than a charge nurse may share the same perspectives of the patient safety culture as staff nurses who were never in charge.

The charge nurse role is separate and distinct from the staff nurse role. Patient safety culture is perceived differently by charge nurses; isolating these differences may help to address the variations in nurses' involvement in evidence-based practice guidelines. If the charge nurses are expected to serve as champion of change for effectiveness research initiatives, tailored educational approaches may be necessary based on their length of time as a charge nurse.

## 6. Limitations

First, this was a cross-sectional study, and the causal direction of the variables used in the study cannot be determined. Second, the study was conducted in a single, large academic medical center. The lack of designated charge nurse positions in this study setting made it difficult to truly test for differences in charge experience as nurses moved in and out of that role. Third, the use of a convenience sample is often associated with selection bias that may limit the generalizability of the results. For example, a greater percent of nurses with a bachelor's degree participated in the study. Future studies that use a probability sample design may increase the likelihood that the sample is representative of the population of charge nurses from which the sample was drawn.

## 7. Implications

Researchers and nurse managers who are interested in improving the safety culture and effectiveness research initiatives may benefit from assessments of the effects of contextual factors on implementation [48]. Understanding that differences in perceptions exist between nurses with varying levels of charge nurse experience may shed light on the mixed results found in the study by Rich et al. [49] about the use of opinion leaders and change champions for the uptake of practice guidelines. Proper training of healthcare team members is essential to develop effective partnerships for research implementation [50]. The success of utilizing evidence-based practice relies on the use of care providers members who serve to clarify program objectives and motivate colleagues [22, 50]. Nurse champions are most effective when the implementation strategy is tailored to meet the organizational contextual need [1]. The charge nurses were less positive than noncharge nurses about perceptions of patient safety culture. Charge nurses may be able to provide nuanced insights about the state of the patient safety culture, which can be explored further by including them in discussions about new initiatives. The effective use of charge nurses as change champions in implementation studies may necessitate their participation in the planning stages for the implementation of new practice guidelines and training about implementation strategies.

## 8. Conclusion

This study highlights the importance of charge and noncharge nurses' perceptions of patient safety culture. Recognition of the importance of the charge nurse role in the assessment of patient safety culture may serve to improve the effective use of nurses as change champions. Future studies should assess the association of the implementation of evidence-based practice guidelines and perception of patient safety culture among nurses.

## Figures and Tables

**Table 1:** Sample characteristics.

Variable	Frequency	
	*N*	Percentage
Shift normally worked (*n* = 333)		
Day	114	**34.2**
Night	113	**33.9**
Shift rotators	106	**31.8**
Number of years as registered nurse on current unit (*n* = 373)		
Less than 1 year	33	**8.8**
1 to 5 years	220	**59.0**
6 or more years	120	**32.2**
Highest degree obtained (*n* = 375)		
Diploma and associate	144	**38.4**
Baccalaureate	205	**54.7**
Masters	26	**6.9**

**Table 2:** Charge nurse characteristics.

Variable	*N*	Percentage
Charge nurse experience (*n* = 374)		
(1) some charge	215	57.5
(a) permanent charge	23	**6.1**
(b) relief charge	192	**51.3**
(2) no charge (staff nurse)	159	**42.5**
Percentage shifts worked incharge in the past month (*n* = 207)		
<25% of shifts worked	92	44.4
>25% of shifts worked	115	**55.5**
Number of years as a charge nurse on current unit (*n* = 228)		
Less than 1 year	30	13.2
1 to 5 years	114	50.0
More than 5 years	84	**36.8**
Shadow-charge orientation (*n* = 228)		
None	17	7.5
1-2 shifts	144	**63.2**
3 or more shifts	67	**29.4**

**Table 3:** *t*-tests for charge nurse experience and AHRQ perception of patient safety culture.

Outcome*	No charge (*n* = 159)	Some charge (*n* = 215)		
	Mean (SD)	Mean (SD)	*t*-value	P**
Overall perceptions of safety	3.46 (0.61)	3.27 (0.63)	2.86	.01
Number of events reported within the last 12 months	2.06 (0.70)	2.31 (0.70)	−3.35	.01

*Outcome was rated from 1 (strongly disagree) to 5 (strongly agree).

**Two-tailed *P* value.

Table only includes significant findings, full results can be obtained from corresponding author.

**Table 4:** Chi-square for charge nurse experience and AHRQ perception of patient safety culture.

Variable	No event	1-2 events	3–21 events	Total
None	32 (19.9)	84 (52.2)	45 (28.0)	161
Less than 25%	11 (12.2)	42 (46.7)	37 (41.1)	90
More than 25%	18 (15.7)	42 (36.5)	55 (47.8)	115
Total	61	168	137	366

*X* ^2^(4) = 13.240; *P* = .010.
